# Intrauterine exposure to low-dose DBP in the mice induces obesity in offspring via suppression of UCP1 mediated ER stress

**DOI:** 10.1038/s41598-020-73477-3

**Published:** 2020-10-01

**Authors:** Huan Li, Jianqiao Li, Zhenting Qu, Honghao Qian, Jing Zhang, Hongyan Wang, Xiaolei Xu, Shengyuan Liu

**Affiliations:** 1 School of Public Health, Beihua University, Jilin, 132013 China; 2 Jilin Combine Traditional Chinese and Western Hospital, Jilin, 132012 China; 3 Shenzhen Nanshan Center for Chronic Disease Control, Shenzhen, 518054 China

**Keywords:** Pathogenesis, Risk factors, Endocrine system and metabolic diseases

## Abstract

Dibutyl phthalate (DBP) is recognized as an environmental endocrine disruptor that has been detected in fetal and postnatal samples. Recent evidence found that in utero DBP exposure was associated with an increase of adipose tissue weight and serum lipids in offspring, but the precise mechanism is unknown. Here we aimed to study the effects of in utero DBP exposure on obesity in offspring and examine possible mechanisms. SPF C57BL/6J pregnant mice were gavaged with either DBP (5 mg /kg/day) or corn oil, from gestational day 12 until postnatal day 7. After the offspring were weaned, the mice were fed a standard diet for 21 weeks, and in the last 2 weeks 20 mice were selected for TUDCA treatment. Intrauterine exposure to low-dose DBP promoted obesity in offspring, with evidence of glucose and lipid metabolic disorders and a decreased metabolic rate. Compared to controls, the DBP exposed mice had lower expression of UCP1 and significantly higher expression of Bip and Chop, known markers of endoplasmic reticulum (ER) stress. However, TUDCA treatment of DBP exposed mice returned these parameters nearly to the levels of the controls, with increased expression of UCP1, lower expression of Bip and Chop and ameliorated obesity. Intrauterine exposure of mice to low-dose DBP appears to promote obesity in offspring by inhibiting UCP1 via ER stress, a process that was largely reversed by treatment with TUDCA.

## Introduction

Obesity is a metabolic disorder disease characterized by energy imbalance and excessive accumulation of fat. The mechanisms that cause the disease, however, have not yet been fully elucidated. It is known that genetic factors, poor diet and a sedentary lifestyle are the main causes of obesity, but only 16% of obese individuals are genetically obese. One hypothesis proposes that environmental factors are the main cause of obesity, although the exact environmental factors that lead to obesity have not been clarified. The obesogens hypothesis suggests that endocrine disrupting chemicals (EDCs) in the environment are the main cause of obesity^1,2^.

Phthalate esters (PAEs), which are widely used in the manufacturing of plastics have been implicated as EDCs, and studies have suggested that low-dose PAEs exposure may be associated with obesity. A cross-sectional study of 5149 subjects conducted by the National Health and Nutrition Examination Survey (NHANES) found a statistically significant association of Body Mass Index (BMI) and waist circumference with phthalates^3^. In a prospective study of 70-year-old adults from Uppsala University in Sweden, insulin resistance was found to be closely correlated with exposure to phthalate metabolites^4^. This study revealed that phthalate metabolites may interfere with glucose metabolism by Peroxisome proliferator-activated receptors (PPARs) pathway. There is also evidence that Mono(2-ethylhexyl) phthalate (MEHP) interferes with the biological transformation of adipose tissue, disrupts the endocrine hormone system and causes the dysregulation of the hypothalamic–pituitary–adrenal control system to promote the formation of fat through a variety of biological pathways, including interference with steroid or thyroid hormones and activation of PPARs^5^.

Exposure to endocrine disruptors during the window of ontogeny can lead to lipogenesis and obesity. Decock et al. found that exposure to EDCs in early life was associated with obesity in adulthood^6^. In utero exposure to di-(2-ethylhexyl) phthalate (DEHP) promotes local adipose tissue inflammation in adult male offspring^7^. Similar observations were made by Maresca et al. in an urban population cohort, suggesting that pre-natal DEHP exposure is associated with increases in early childhood BMI, waist circumference and body fat content^8^. Meta-analysis showed that early exposure to DEHP could increase obesity in rodents^9^. A generalized linear model analyzed the relationship between DEHP metabolites and obesity-related markers, including serum levels of leptin, total cholesterol (TC) and triglycerides (TG), and found that early exposure to DEHP could affect these indicators, leading to weight changes^10^. Studies on chronic diseases in rats have confirmed that dibutyl phthalate (DBP) significantly increased the incidence of chronic diseases, such as hypertension and diabetes, in offspring rats exposed to increased levels of maternal intrauterine plasticizer^11^. However, there are few reports on the effects of DBP exposure during fetal development on obesity. In this study, intrauterine DBP exposure was used to study its effect on offspring obesity.

## Materials and methods

### Animals and treatments

SPF C57BL/6J mice aged 8 weeks were obtained from the Laboratory Animal Center of Jilin University (Changchun, Jilin, China). Mice were fed a standard diet containing (g%): 22.60% protein, 50.87% carbohydrate, 3.37% lipid, 3.33% fibers, 6.88% mineral and 12.95% water, and maintained at 22 ± 1 °C with a 12-h light/dark cycle and food and water ad libitum. The animal experiment was performed in accordance with the National Institutes of Health Guide for the Care and Use of Laboratory Animals and was approved by the Animal Care and Use Committee of Beihua University.

The mice were adapted to laboratory conditions for 1 week before the experiments began. Males were placed in the female’s cage (1:1 ratio) overnight for mating. The next morning, vaginal swabs were taken, and pregnancy day 1 was determined by the presence of sperm. Pregnant mice were randomly divided into two groups. Mice in the DBP group were administered 5 mg/kg/day of DBP (analytical grade, purity 99.5%) (Sigma, USA) by a single oral gavage daily from the 12th day of pregnancy to postnatal day 7. Pregnant mice in the control group were gavaged with an equivalent volume of corn oil on the same days. Each group of offspring, including at least 30 male and 30 female mice, were weaned 21 days after birth. All offspring were fed a standard diet during the 21-week duration of the study. During the last 2 weeks of the study period, 20 male and 20 female mice were selected from both the DBP and Ctrl groups and each group was divided into two subgroups, one receiving 14 days of 500 mg/kg i.p. tauroursodeoxycholic acid (TUDCA) (Sao Paulo, Brazil), (subgroups: Ctrl + TUDCA; DBP + TUDCA) and the control group receiving i.p saline (subgroups: Ctrl + saline; DBP + saline). After the total 21 weeks and an overnight 12 h fast, all animals were sacrificed and samples were taken of blood and of adipose tissue deposits in the testes, liver and pancreas.

### Body composition measurements

At the end of the 21 week trial, the body composition of mice was analyzed precisely for total body fat and lean mass using the small animal body composition analyzer (Minispec LF-50, Bruker, Germany).

### Glucose and insulin tolerance tests

At the end of week 21, mice were fasted for either 18 h for a glucose tolerance test or 6 h for an insulin tolerance test. Blood was acquired from the tail vein and the fasting blood glucose level were measured (time 0) using One-Touch Ultraglucometers (Life Scan). The animals were then given either 2 g/kg glucose or 0.75 U/kg insulin (Regular Humulin, Eli Lilly and Company) by intraperitoneal injection. Blood glucose curves were drawn based on the glucose in blood samples drawn at 15 min, 30 min, 60 min, and 120 min post injection, and the area under the curve (AUC) was calculated.

### Metabolic cage studies

Individual animals were housed in cages with a 12 h dark/light cycle at room temperature (22 ± 1 °C). Basal food and water intake, urine and feces elimination, oxygen consumption (VO2), production of carbon dioxide (VCO_2_), and locomotor activity were determined during this period by the TSA calorimetric system (TSA System, Germany), and Energy Expenditure (EE) and the Respiratory Quotient (RQ) were calculated using these parameters. The temperature was measured in the anus.

### Serum and liver chemistries

Blood samples were centrifuged at 3000 rpm for 10 min to obtain the serum, and liver tissues were homogenized. Fasting serum insulin and fasting serum leptin were measured with ELISA kits from Dingguo Changsheng Biotechnology (Beijing, China). The serum levels of TG, TC, free fatty acids (FFA), hepatic TG, and hepatic TC were determined using corresponding assay kits (Nanjing Jiancheng Bioengineering Institute, Nanjing, Jiangsu, China).

### Histological analysis

Tissues were fixed in 4% paraformaldehyde (pH 7.4) for 24 h at room temperature, embedded in paraffin, and sectioned into five-micrometer-thick slices. The sections of WAT and the pancreas were stained with hematoxylin and eosin (Dingguo Changsheng Biotechnology, Beijing, China), and the sections of liver were stained with oil red O (Wuhan Goodbio technology Co., Ltd., Wuhan, China). Images were taken with a digital camera (DP20, Olympus, Tokyo, Japan) to assess tissue histopathology.

### Real-time PCR to measure expression

Total mRNA of brown adipose tissue (BAT) was isolated with RNAiso plus (TaKaRa Dalian Biotechnology, Dalian, China). Reverse transcription was performed by Prime Script RT reagent Kit (TaKaRa Dalian Biotechnology, Dalian, China). Real-time quantitative PCR using SYBR Premix Ex Taq Mix (TaKaRa Dalian Biotechnology, Dalian, China) was carried out in an ABI Q6 Real-time PCR system (ABI, Carlsbad, CA, USA). The total reaction volume was 7 µl, including 0.25 µl of each primer (10 µM), 2 µl of tenfold diluted cDNA, 3.5 µl of SYBR Premix Ex Taq, and 1 µl of RNase-Free H_2_O. The specific reaction conditions were: 2 min at 95 °C, followed by 40 cycles of 15 s at 95 °C and 1 min at 60 °C. The results were normalized toβ-actin levels (2^−ΔΔCt^ Method). The primers used were obtained from Sangon Biotech (Shanghai, China) and are listed in Table 1.

**Table 1:** Primer sequences used for RT-PCR.

Gene	Primer sequences (5′-3′)	Length
**UCP1**		
F	CACCATGGTGAACCCGACAACTTCC	25
R	TTATGTGGTACAATCCACTG	20
**Pgc-1α**		
F	AACAATGAGCCTGCGAAC	18
R	CCTCGTTGTCAGTGGTCA	18
**Prdm16**		
F	CAGCACGGTGAAGCCATTC	19
R	GCGTGCATCCGCTTGTG	17
**Cidea**		
F	AGGCCGTGTTAAGGAATCTG	20
R	CCCAGTACTCGGAGCATGTA	20
**Bip**		
F	TGAAACTGTGGGAGGAGTCA	20
R	TTCAGCTGTCACTCGGAGAA	20
**Chop**		
F	CTGCCTTTCACCTTGGAGAC	20
R	CGTTTCCTGGGGATGAGATA	20
**β-actin**		
F	CATCCGTAAAGACCTCTATGCCAAC	25
R	ATGGAGCCACCGATCCACA	19

### Western blot analysis

BAT was separated, frozen in liquid nitrogen and stored at − 80 °C until use. For western blot analysis, the samples were homogenized in RIPA buffer containing 0.1% PMSF (KeyGEN BioTECH, Nanjing, China), centrifuged (12,000×*g*, 15 min, 4 °C), and the supernatant was collected. The protein content was determined using a BCA kit (Thermo Fisher Scientific, San Jose, CA, USA). Equal amounts of protein (60 µg/lane) were separated on 8% SDS-PAGE gels and then transferred onto PVDF membranes. After blocking with 5% skimmed milk for 2 h at room temperature, the membranes were incubated with the following primary antibodies:β-actin (1:500, Santa Cruz, CA, USA); Uncoupling protein 1(UCP1) (1:1000, Santa Cruz); Binding immunoglobulin protein (Bip) (1:1000, Santa Cruz); CCAAT/enhancer-binding protein homologous protein (Chop) (1:1000, Santa Cruz) in TBS-T containing 3% BSA overnight at 4 °C. The membranes were then incubated with HRP-conjugated secondary antibody (1:5000 dilution, Dingguo Changsheng Biotechnology, Beijing, China) at 37 °C for 2 h. After three washes in TBS-T, the images were detected using a chemiluminescent detection system (Tanon Image System Ver.5200, Shanghai, China).

### Statistical analysis

All data were presented as the mean ± Std Dev and statistical significance determined by one-way analysis of variance (ANOVA) followed by Fisher’s least significant difference (LSD) test, using SPSS statistical software (SPSS 13.0 software, SPSS Inc, Chicago, IL, USA). Statistical significance was considered to be *P* < 0.05.

## Results

### Adipose tissue deposits

**Figure 1:**
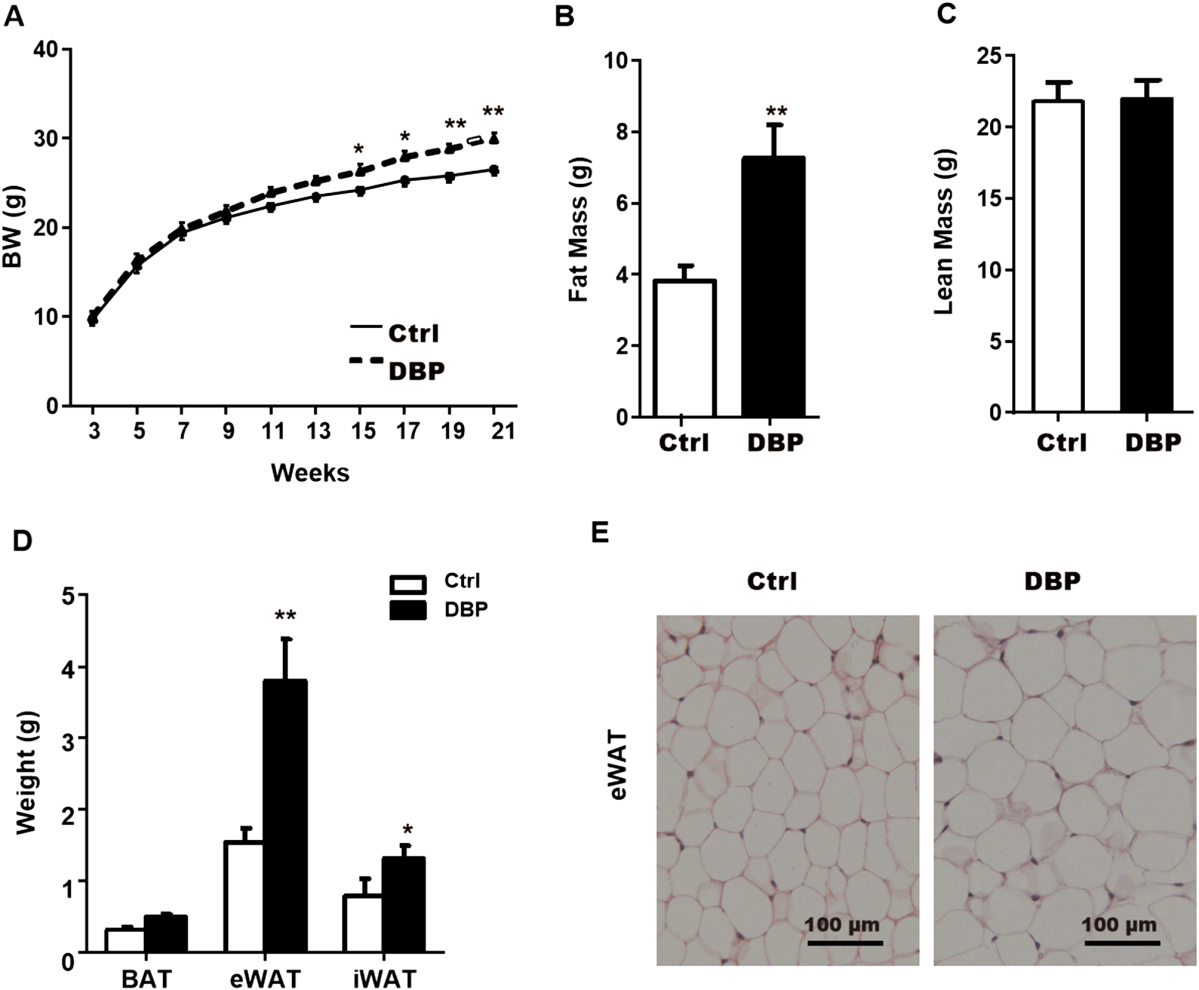
Effect of in utero DBP exposure on body weight and adipose tissue in offspring. (**A**) Body weight (BW) during 3–21 weeks in offspring. (**B**) Fat mass at the 21 week. (**C**) Lean mass at the 21 week. (**D**) Adipose tissue weight from different depots. (**E**) WAT from Ctrl and DBP mice stained with hematoxylin–eosin (H&E), Scale bars = 100 μm. Data are expressed as the mean ± SD, n = 10 per group, *p < 0.05, **p < 0.01 versus the Ctrl group. *BW* body weight, *Ctrl* control group, *DBP* DBP group, *WAT* White adipose tissue, *BAT* brown adipose tissue, *eWAT* epididymal WAT, *iWAT* inguinal WAT, *H&E* hematoxylin and eosin.

The growth and development of all mice were normal throughout the experimental period, but the body weights in the DBP group were significantly higher than those in the Ctrl group from 15 to 21 weeks (Fig. 1A). Compared with the Ctrl group, at 21 weeks the fat mass, epididymal white adipose tissue (eWAT), and inguinal white adipose tissue (iWAT) in the DBP group were significantly increased (Fig. 1B,D). While lean mass and brown adipose tissue (BAT) presented no statistical difference between the Ctrl and DBP groups at 21 weeks (Fig. 1C,D). H&E staining showed that the volume of the adipocytes that constitute the eWAT in the DBP group was significantly larger than in the Ctrl group (Fig. 1E).

### Glucose metabolic disorder

**Figure 2:**
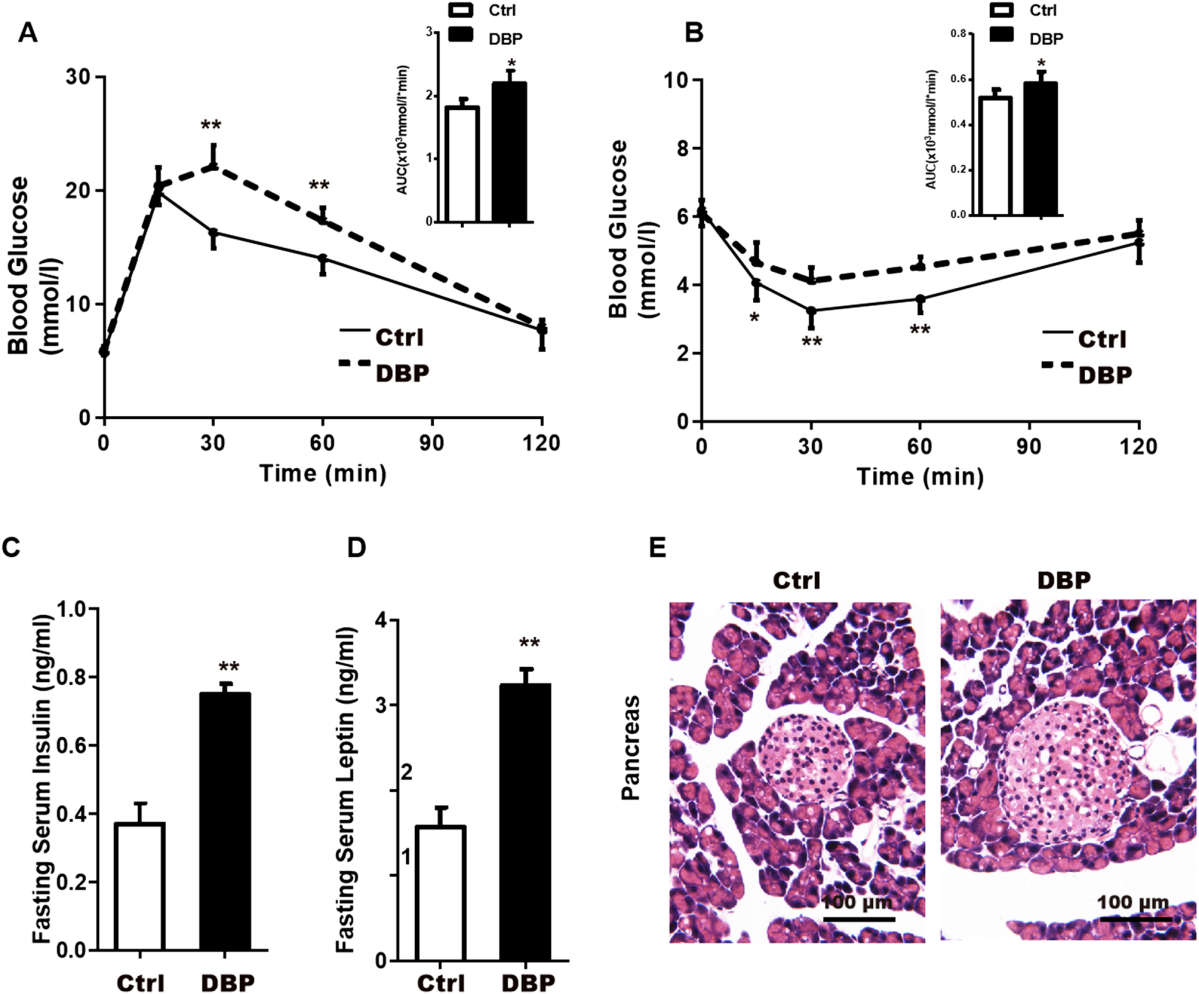
Intrauterine exposure to DBP induces glucose metabolic disorder in offspring. (**A**) Glucose tolerance and the area under the curve (AUC) at 21 weeks. (**B**) Insulin sensitivity and the area under the curve at 21 weeks. (**C**) The levels of fasting serum insulin. (**D**) The levels of fasting serum leptin. (**E**) Hematoxylin–eosin (H&E) staining of the pancreas tissue, Scale bars = 100 μm. Data are expressed as the mean ± SD, n = 10 per group, *p < 0.05, **p < 0.01 versus the Ctrl group.

The glucose tolerance test showed that the blood glucose AUC was significantly higher in the DBP group compared to the Ctrl group, and was higher at 30 min and 60 min, as shown in Fig. 2A. In addition, the insulin tolerance test showed higher blood glucose levels in the DBP group than in controls at 15 min, 30 min and 60 min after insulin injection (Fig. 2B), indicating that DBP exposure could lead to insulin resistance. In addition, fasting serum insulin and leptin levels were both significantly elevated in the mice exposed to DBP (Fig. 2C,D).

H&E stained sections of the pancreas showed that the arrangement of the islet cells in DBP group was disordered, the boundary was unclear, and the size and shape of the islet nuclei were irregular. In addition, some islet cells in DBP group showed vacuolar degeneration (Fig. 2E).

### Lipid metabolism

**Figure 3:**
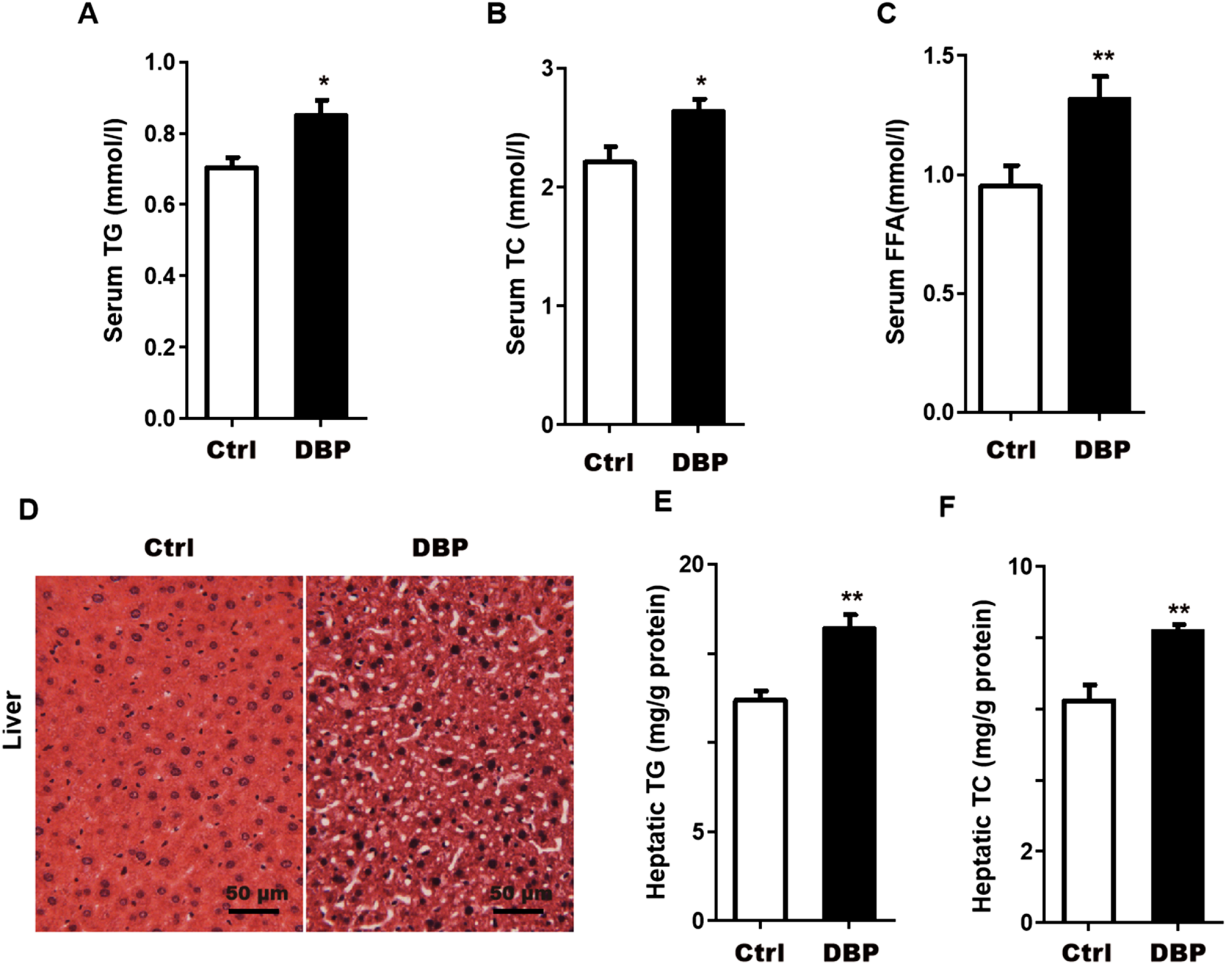
Serum and liver factors associated with obesity and lipid metabolism disorder. (**A**) Serum triglycerides, TG. (**B**) Serum total cholesterol, TC. (**C**) Serum free fatty acid, FFA. (**D**) Lipid droplets in liver slices were observed by oil red O staining, Scale bars = 50 μm. (**E**) Hepatic triglyceride, TG. (**F**) Hepatic total cholesterol, TC. Data are expressed as the mean ± SD, n = 10 per group, *p < 0.05, **p < 0.01 versus the Ctrl group.

To investigate the effect of DBP exposure on lipid metabolism, we measured several relevant biochemical indicators. The results showed that, compared with the Ctrl group, serum TG, TC, and FFA, as well as hepatic TG and TC were significantly increased in DBP group (Fig. 3A–C,E,F). Furthermore, in liver slices stained with oil red O, lipid droplet accumulation was greater in hepatocytes of DBP exposed mice compared to the Ctrl group (Fig. 3D).

### Energy metabolism

**Figure 4:**
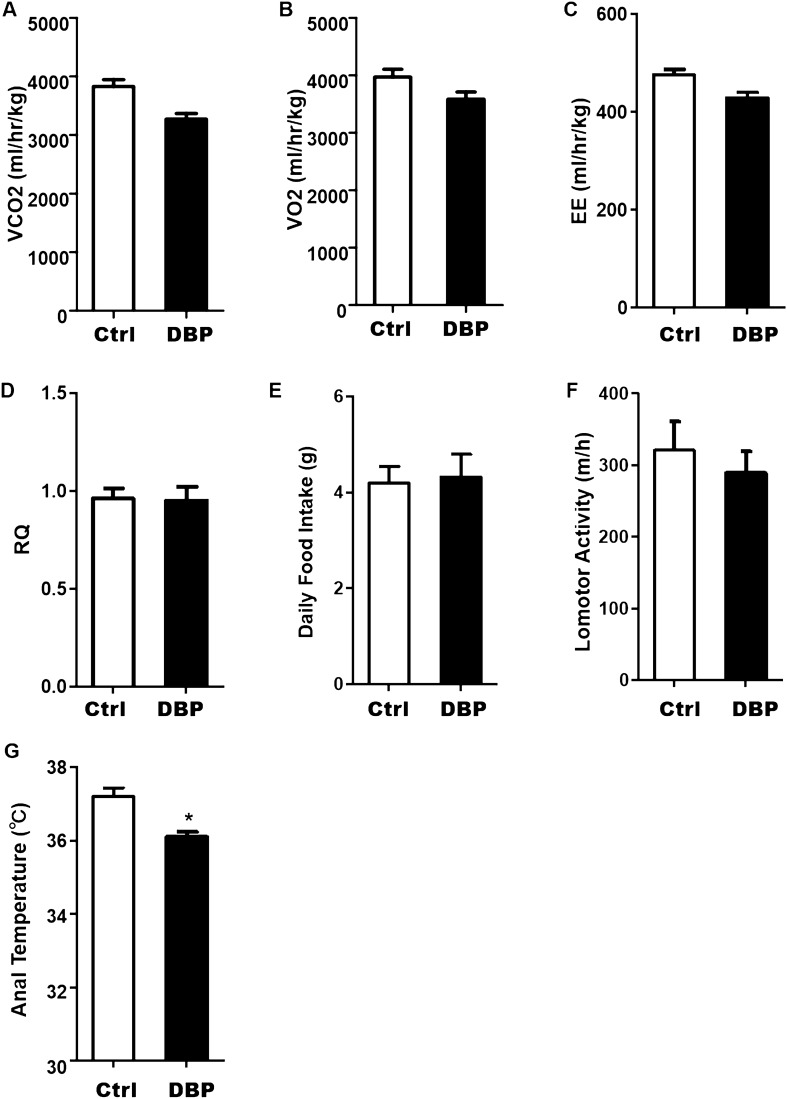
Effect of in utero DBP exposure on energy metabolism in offspring. (**A**) Production of carbon dioxide. (**B**) Oxygen consumption. (**C**) Energy expenditure. (**D**) Respiratory Quotient (VCO_2_/VO_2_). (**E**) Daily food intake. (**F**) Lomotor activity. (**G**) Anal temperature. Data are expressed as the mean ± SD, n = 10 per group, *p < 0.05 versus the Ctrl group.

To examine whether the increases in body weight and WAT content after in utero DBP exposure offspring were related to food intake or activity, we performed a metabolic cage test. The results presented in Fig. 4A–C,F show that oxygen consumption, carbon dioxide production, energy expenditure, and locomotor activity in DBP group were all slightly lower than in the Ctrl group, but the differences between the two groups were not significant. There were also no statistical differences between the two groups in food intake or RQ (Fig. 4D,E), but the anal temperature was lower in the DBP group (Fig. 4G).

### Inhibition of UCP1 expression and endoplasmic reticulum (ER) stress

**Figure 5:**
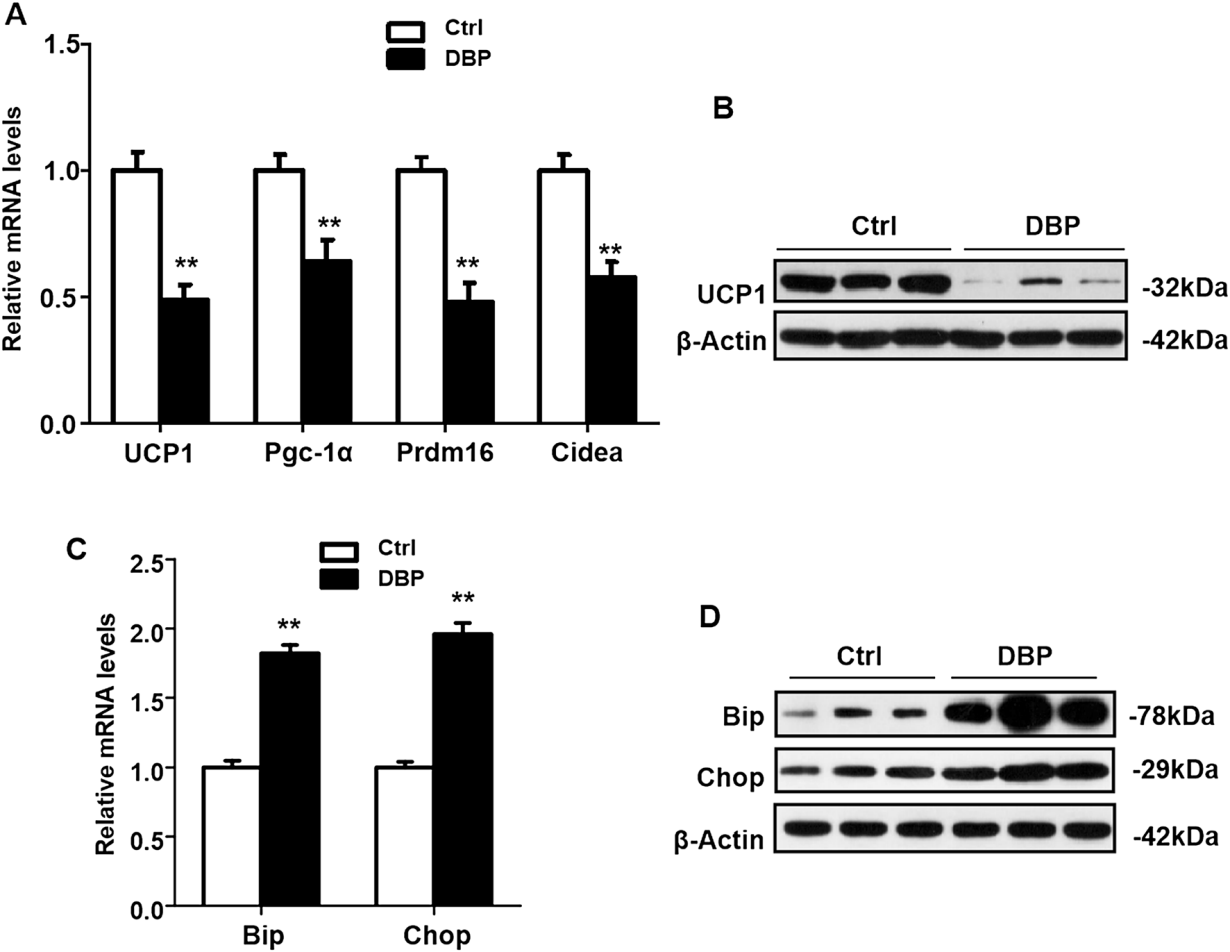
Intrauterine exposure to DBP decreased UCP1 protein expression in offspring. (**A**) The relative mRNA expression levels of UCP1, Pgc-1α, Prdm16 and Cidea were analyzed by RT-PCR. (**B**) The protein levels of UCP1 was assessed by western blot. Their corresponding full-length blots are presented in Supplementary Fig. S1. (**C**) The relative mRNA expression levels of Bip and Chop were analyzed by RT-PCR. (**D**) The protein levels of Bip and Chop was analyzed by Western blot. Their corresponding full-length blots are presented in Supplementary Fig. S2. All data above were expressed as the mean ± SD of three independent experiments. Data are expressed as the mean ± SD, n = 10 per group, **p < 0.01 versus the Ctrl group.

To explore whether UCP1 was involved in the obesity seen in the DBP exposed offspring, the relative mRNA levels of UCP1, Peroxisome proliferator-activated receptor γ coactivator 1α (Pgc-1α), Transcription factor PR domain containing 16 (Prdm16) and Cell-death inducing DNA fragmentation factor-like effector A (Cidea) in mice were analyzed by RT-PCR, and UCP1 protein expression was evaluated by Western blotting. As shown in Fig. 5A,B, the relative mRNA levels of UCP1, Pgc-1α, Prdm16 and Cidea were significantly reduced, and UCP1 protein expression was lower in the offspring with intrauterine DBP exposure. To investigate whether this obesity was associated with ER stress, the expression of Bip and Chop was measured by RT-PCR and Western blotting. The results indicated that intrauterine DBP exposure increased the expression of Bip and Chop, as determined by both relative mRNA and protein levels (Fig. 5C,D).

### TUDCA ameliorated obesity in mice by inhibiting ER stress

**Figure 6:**
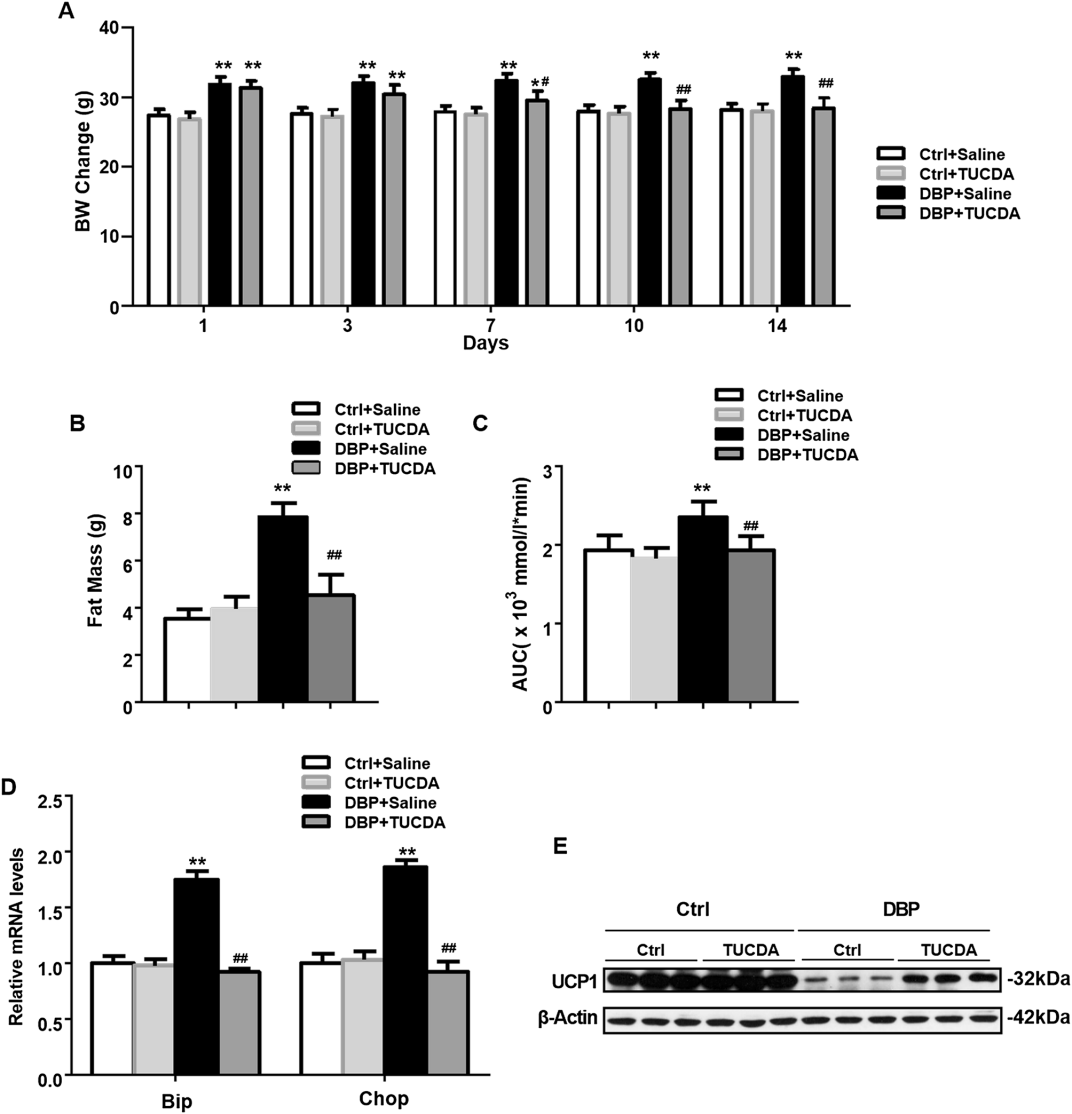
TUDCA treatment inhibited obesity in mice by up-regulating UCP1 expression. (**A**) Body weight change during 1–14 days in offspring. (**B**) Fat mass at the 14 day. (**C**) Area under curve of glucose. (**D**) The relative mRNA levels of Bip and Chop analyzed by RT-PCR. (**E**) Protein levels of UCP1. All data above were expressed as the mean ± SD of three independent experiments. Their corresponding full-length blots are presented in Supplementary Fig. S3. Data are expressed as the mean ± SD, n = 10 per group, *p < 0.05, **p < 0.01 versus the Ctrl + Saline group, ^#^*p* < 0.05, ^##^*p* < 0.01 versus the DBP + Saline group.

To further investigate whether ER stress is involved in DBP-induced obesity,mice were treated with TUDCA. The body weight in DBP exposed mice was significantly greater than in controls over days 1–14 (Fig. 6A), but subsequent treatment with TUDCA (DBP + TUDCA) reduced the body weight in mice from 7 to 14 days, while not altering body weight in the Ctrl + TUDCA mice. Compared to controls, DBP exposed mice had a higher total fat content and greater AUC for blood glucose, but TUDCA treatment (DBP + TUDCA) restored these parameters to levels similar to those found in Ctrl mice. Finally, compared with DBP + saline mice, TUDCA treated mice (DBP + TUDCA) showed decreased relative mRNA levels for Bip and Chop and higher UCP1 protein expression, suggesting that endoplasmic reticulum stress participates in DBP-induced obesity by suppression of UCP1 (Fig. 6D,E).

## Discussion

EDCs can disrupt the programmed function of endocrine signaling pathway and interfere with or block the secretion, synthesis, release, binding, activation, inactivation, and metabolism of normal hormones. The specific manifestations are: disruption of major weight control hormones such as catecholamine, thyroid hormone, estrogen, insulin, growth hormone, leptin, testosterone and corticosteroids; altered hormone sensitivity, especially for dopamine, serotonin, and angiotensin; and disruption of metabolic processes. Together, these effects can lead to tissue and organ damage, especially in nerves and muscles^12–16^, even at low levels of exposure. In our study, pregnant mice were administered DBP was from the 12th day of gestation to the 7th day after birth, a critical period in the differentiation of adipose tissue, reproductive organs and the immune system. At this stage of development, fetuses and newborns are very sensitive to environmental endocrine disruptors^17,18^, which can pass through the maternal blood barriers to reach the placenta and breast milk.

An important theory in the field of environmental endocrinology is the low dose effect of EDCs, which can show a nonmonotonic relation of “U” or inverted “U”. For example, low-dose prenatal and postnatal exposure to Bisphenol A (BPA) (1 mg/L of drinking water) induced obesity and hyperlipidemia in offspring mice, while fat accumulation was not obvious with high-dose exposure to BPA (10 mg/L drinking water)^18^. Similarly, increased weight and fat gain were seen in the male offspring of mice treated with low doses of MEHP (0.05 mg/kg b.w.), but not with high doses (0.5 and 0.25 mg/kg b.w.)^19^. In addition, a recent study showed^20^ that low doses of genistein led to fat accumulation in adipose tissues, but high doses inhibited fat accumulation in adipose tissues, especially in men. Moreover, the accumulation of fat caused by genistein was associated with insulin resistance. Based on these prior studies, we used a low dose of DBP (1/20 of mouse oral LD_50_) for intrauterine exposure in this study.

As phthalate metabolites are known ligands of PPARs^21^, they can affect the glucose balance in vivo*.* By damaging the PPAR signal bypass, they are involved in different stages of glucose homeostasis, participate in glucose metabolism, affect insulin secretion^22^ the level of fat circulation, and the quantities of visceral and subcutaneous fat deposits^23^. Measurements of weight, abdominal fat weight, and fat cell volume are the principal criteria for judging obesity. In the present study, offspring with intrauterine exposure to DBP displayed significantly greater gains in body weight from 15 to 21 weeks postnatal compared with mice in the control group. Although there was no significant change in BAT between the two groups, the weights of the eWAT and iWAT were significantly increased in the DBP group, with almost twice as much eWAT, which represents visceral fat, than was seen in the non-exposed mice.

Leptin is the product of the obese gene and plays an important role in regulating appetite, feeding and energy expenditure^24^. The incomplete transport of leptin through the blood–brain barrier, the occurrence of mutations in the leptin receptor or the impairment of the leptin signaling are some of the known causes that can lead to leptin resistance, which is manifested by the presence of hyperleptinemia in the body^25^. We found that, compared with the Ctrl mice, the secretion of leptin was increased in the DBP intrauterine exposed offspring, suggesting that intrauterine DBP exposure could cause leptin resistance. Body weight and body fat content are balanced by energy expenditure and caloric intake, but we found no significant difference in food intake between the DBP and Ctrl groups. However, while the activity level of mice in DBP group was only slightly decreased, the body temperature of the DBP exposed mice was lower. Analysis of metabolic cage results showed that the production of carbon dioxide, energy expenditure and the locomotor activity of the DBP exposed mice were slightly lower than in the control group, but the differences were not significant. This suggests that the increase in the body weight and WAT content of DBP exposed mice was not caused by excessive food intake or decreased activity, but rather appears to be related to a decreased metabolic rate.

The fat tissue of DBP exposed mice showed a significant increase in adipocyte volume, which is a further indication of an increased in the stored lipids in fat cells. The liver plays a central role in fat uptake and oxidation, as well as in sugar and fatty acid metabolism and cholesterol generation^26^. In our study, we measured fasting blood-glucose, serum levels of TG, TC and insulin, hepatic TG and TC levels. Compared with the control group, fasting blood glucose and insulin levels were higher in the in DBP exposed group, suggesting that intrauterine exposure to DBP may induce disorders of glucose metabolism in offspring. The levels of cholesterol and triglyceride in the serum and liver of DBP exposed mice were also significantly higher than in the Ctrl group, indicating that the exposed mice not only developed obesity but also dyslipidemia. These results are similar to the metabolic effects on offspring reported for exposure to DEHP and MEHP, analogues of DBP. Mangala et al. treated pregnant rats with a low concentration (1 mg/kg/day) of DEHP and found that offspring had significantly increased fasting blood glucose levels as well as altered glucose intake and absorption^27^. Hao et al. found that prenatal exposure to MEHP in mice increased the blood glucose of male offspring by about 79%^28^. Our study thus confirms that that phthalates are associated with chronic endocrine diseases.

Adipose tissue was divided into two categories: WAT, which is distributed under the skin and in areas such as mesentery; and BAT which is found in small amounts around the neck, between the shoulders, and around the ribs^29^. WAT is a major contributor to obesity. It contains large fat droplets that store excess energy, mainly in the form of triglycerides, and secretes numerous adipokines such as leptin and adiponectin, which regulate the body’s energy metabolism. BAT adipocytes contain many small fat droplets and are rich in organelles and cytoplasm, especially mitochondria. The mitochondria of brown fat cells express specific UCP1, which may have an anti-obesity effect because it can oxidize and decompose fat^30^. UCP1 is a protein that is essential for brown fat cells to perform their heat-producing functions^31^, and the expression of Pgc-1α, Prdm16 and Cidea in BAT has been associated with fat metabolism. Pgc-1αis a key transcriptional regulator of heat production in BAT, and regulates expression of UCP in BAT. The abnormal expression of Pgc-1αand UCP1 in brown fat cells appears to be involved in the generation of obesity. In rats with ob/ob, db/db, and fa/fa genetic obesity, the expression of Pgc-1α and UCP1 in brown fat cells was decreased, along with reduced activity of fatty acid oxidation enzymes and energy metabolism^32^. Rich Prdm16 in BAT promotes brown fat maturation by increasing mitochondrial gene expression, mitochondrial density, adipocyte heat production and energy consumption, which can also increase the expression of UCP1 Moreover, Prdm16 can inhibit certain cytokines and hormones encoded by WAT cells, thereby further affecting lipid metabolism^33^. In wild-type mice, Cidea is mainly expressed in BAT, and molecular mechanism studies indicated that Cidea may regulate fat metabolism by regulating the activity of UCP1 in BAT^34^. Studies have found that energy metabolism and fat decomposition were accelerated and body fat accumulation was reduced in Cidea gene knockout mice, allowing them to resist the obesity normally induced by a high-fat diet. The accumulation of lipids in fat tissue was significantly reduced, while lipolysis was significantly increased in Cidea gene knockout mice. In addition, Cidea knockout mice showed increased glucose tolerance, significantly faster glucose absorption and increased insulin sensitivity^35^. In our study, the mRNA levels of UCP1, Pgc-1α, Prdm16 and Cidea in were lower in the mice with intrauterine DBP exposure than in the non-exposed mice, and the protein level of UCP1 was also decreased. This suggests that by decreasing the expression of Pgc-1α, Prdm16 and Cidea, DBP exposure reduced the levels of UCP1 and lipid degradation and accelerated the accumulation of fat, thus resulting in obesity.

The endoplasmic reticulum is a dynamic membranous organelle with a variety of functions, including lipid synthesis, protein modification, folding, and some intracellular signal transduction. Dysfunctional, misfolded or unfolded proteins accumulate in the endoplasmic reticulum, inducing endoplasmic reticulum stress^36^. Recent studies have shown that the overload of free fatty acids in obese patients leads to the increased production of oxidizing elements that interfere with the correct folding, processing and maturation of proteins in the ER^37^. ER stress was increased in the adipose tissue of obese and insulin resistant patients, and was decreased in adipose tissue and liver after weight loss^38^. Sharma et al. found a significant increase in the expression of endoplasmic reticulum stress related genes in the subcutaneous adipose tissue of obese and overweight people, which was related to body fat content and body mass index. Many studies have shown that ER stress markers can be detected in adipose tissue, liver and hypothalamus of obese mice, suggesting ER stress in these tissues^39^. Inhibition of ER stress level in the insulin target tissues of obese mice can improve their insulin sensitivity and glucose metabolic status^40^, indicating that ER stress plays a role in the development of obesity. Studies have reported that DBP induced male reproductive toxicity through ER stress, which was closely related to oxidative stress in testicular spermatogenic cells^41^. After exposure to DBP, the spermatogenic cells of rat testicles showed ER swelling and expansion, which is characteristic of ER stress. Moreover, studies have shown that DEHP can induce ER stress in mammalian cells^42,43^. All of these findings suggest that ER stress could play an important role in the reproductive toxicity induced by DBP. Therefore, we hypothesized that DBP could also affect nutrient metabolism through ER stress.

When ER stress occurs, the expression of the upstream molecule Bip and the downstream benefit molecule Chop are increased, so the levels of both are often used as criteria for evaluating ER stress^44^. Some studies have shown that UCP1 activity in adipose tissue was regulated by ER stress. Yuliana et al. found that ER stress negatively regulates UCP1 in mice iWAT^45^. Further investigation showed that the regulatory effect of ER stress on UCP1 expression is accomplished via suppression of the peroxisome proliferator-activated receptor γ (Pparγ) in mice beige adipocytes. Okla et al. found that ER stress inhibited UCP1 gene expression and lead to mitochondrial dysfunction in mature human adipocytes^46^. However, there are also reports that the levels of Bip and Chop mRNA expression of BAT increased in UCP1−/− mice, suggesting that UCP1 deficiency promotes ER stress^47^. To investigate whether intrauterine exposure to DBP can induce ER stress and cause obesity in offspring, proteins were extracted from the adipose tissue of mice, and western blot analysis showed that the expression of ER stress markers Bip and Chop was significantly increased in the DBP group compared to the controls. This suggests that DBP-induced obesity might be related to the activation of ER stress in the adipose tissue of mice.

Recent studies have shown that reactive protein chaperone 4-butyrate (4-PBA) and TUDCA promote protein folding in the ER and thereby maintain protein homeostasis^48,49^. Other studies have shown that chaperones can reduce leptin resistance caused by ER stress in vitro and increase leptin sensitivity in obese mice^50^. In obese mice, 4-PBA can reduce ER stress-mediated leptin resistance, and TUDCA can decrease insulin resistance in adipose tissue of obese mice, maintain blood glucose homeostasis, increase insulin sensitivity and reduce fatty liver disease by reducing ER stress without affecting body weight^51^. To further verify whether ER stress is involved in DBP-induced obesity, we treated some of the BDP exposed mice with TUDCA. We found that the expression of ER stress markers Bip and Chop in DBP exposed mice was significantly reduced by treatment with TUDCA, presumably by inhibiting ER stress. Moreover, we found decreases in total fat content and blood glucose AUC, and increases inthe expression of UCP1 in DBP exposed mice treated with TUDCA. It therefore appears that ER stress is involved in lipid metabolism, and perhaps related to the levels of UCP1.

In conclusion, intrauterine exposure to low-dose DBP could induce ER stress, which inhibits the expression of UCP1, thereby decreasing the energy consumption by BAT, and affecting the metabolism of lipids and sugars, eventually leading to obesity in offspring. Therefore, DBP may be a potential chemical inducer of obesity and related metabolic diseases.

This study has some limitations. Firstly, further study is needed to examine the exact mechanisms through which intrauterine exposure to low dose DBP induces ER stress in offspring. Secondly, it remains to be confirmed whether ER stress inhibits UCP1 through direct or indirect pathways. Thirdly, in vitro experiments are needed to define the molecular mechanisms involved in the metabolic dysregulation associated with DBP exposure.

## Supplementary information

Supplementary Information.
