# Supplementary material for: Intrauterine exposure to low-dose DBP in the mice induces obesity in offspring via suppression of UCP1 mediated ER stress
Source: Sci Rep. 2020 Oct 1;10:16360. doi: 10.1038/s41598-020-73477-3 (PMC7529907; doi:10.1038/s41598-020-73477-3)
Supplement: Supplementary file 1 — Supplementary Information. [file 41598_2020_73477_MOESM1_ESM.pdf]

Intrauterine exposure to low-dose DBP in the mice induces obesity in offspring via suppression of UCP1 mediated ER stress.

Huan Li[a#], Jianqiao Li[a#], Zhenting Qu[b], Honghao Qian[a]， Jing Zhang[a]， Hongyan Wang[a]， Xiaolei Xu[a*], Shengyuan Liu[c*]

[a] School of Public Health, Beihua University, Jilin 132013, China;

[b]Jilin combine traditional Chinese and western hospital, Jilin 132012, China;

[c]Shenzhen Nanshan Center for Chronic Disease Control, Shenzhen 518054, China;

[#]These authors contributed equally to this work as the first author.

[*]These authors contributed equally to this study as the last author.

Corresponding author:

To whom correspondence should be addressed. liushenglb@126.com;

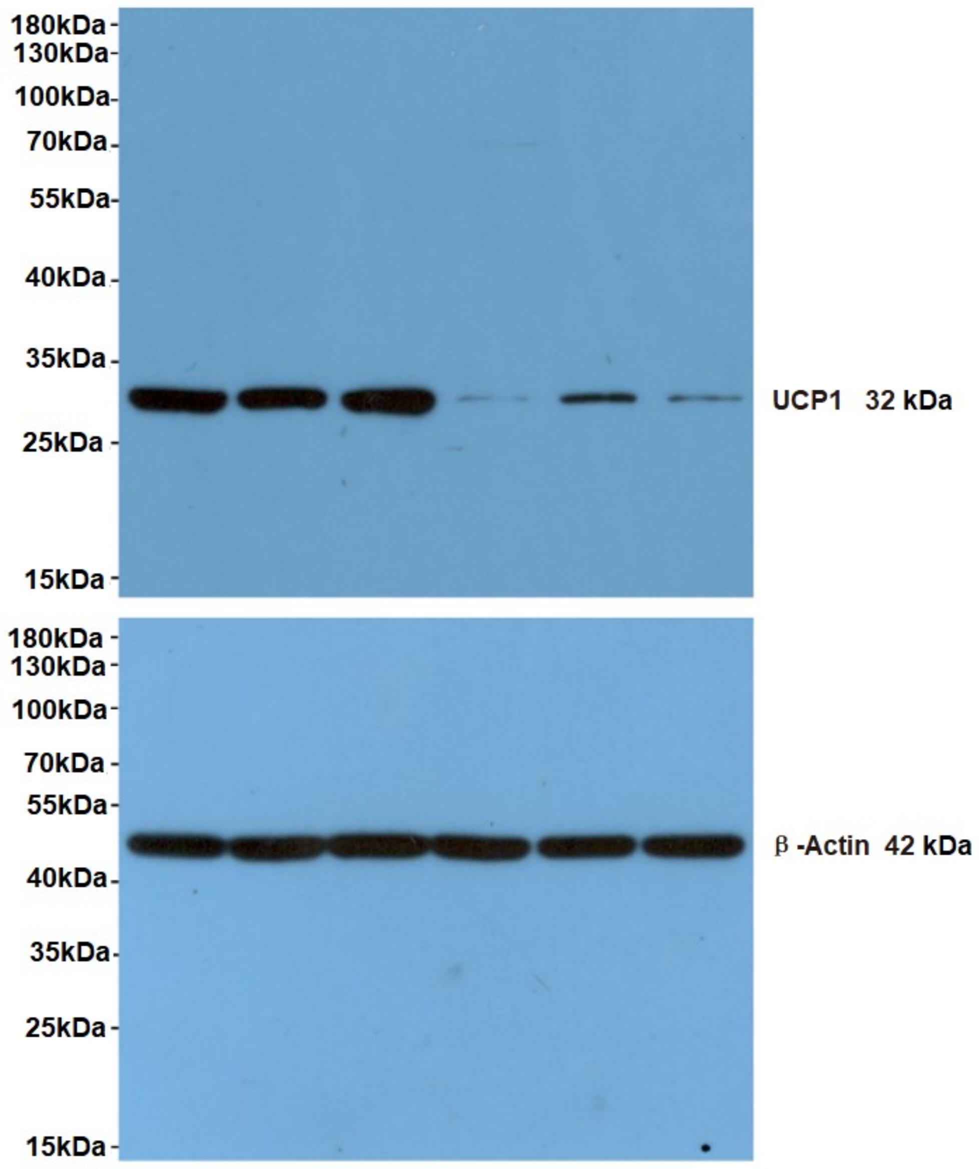

Figure S1: The full length western blots for the UCP1 proteins in Fig. 5.

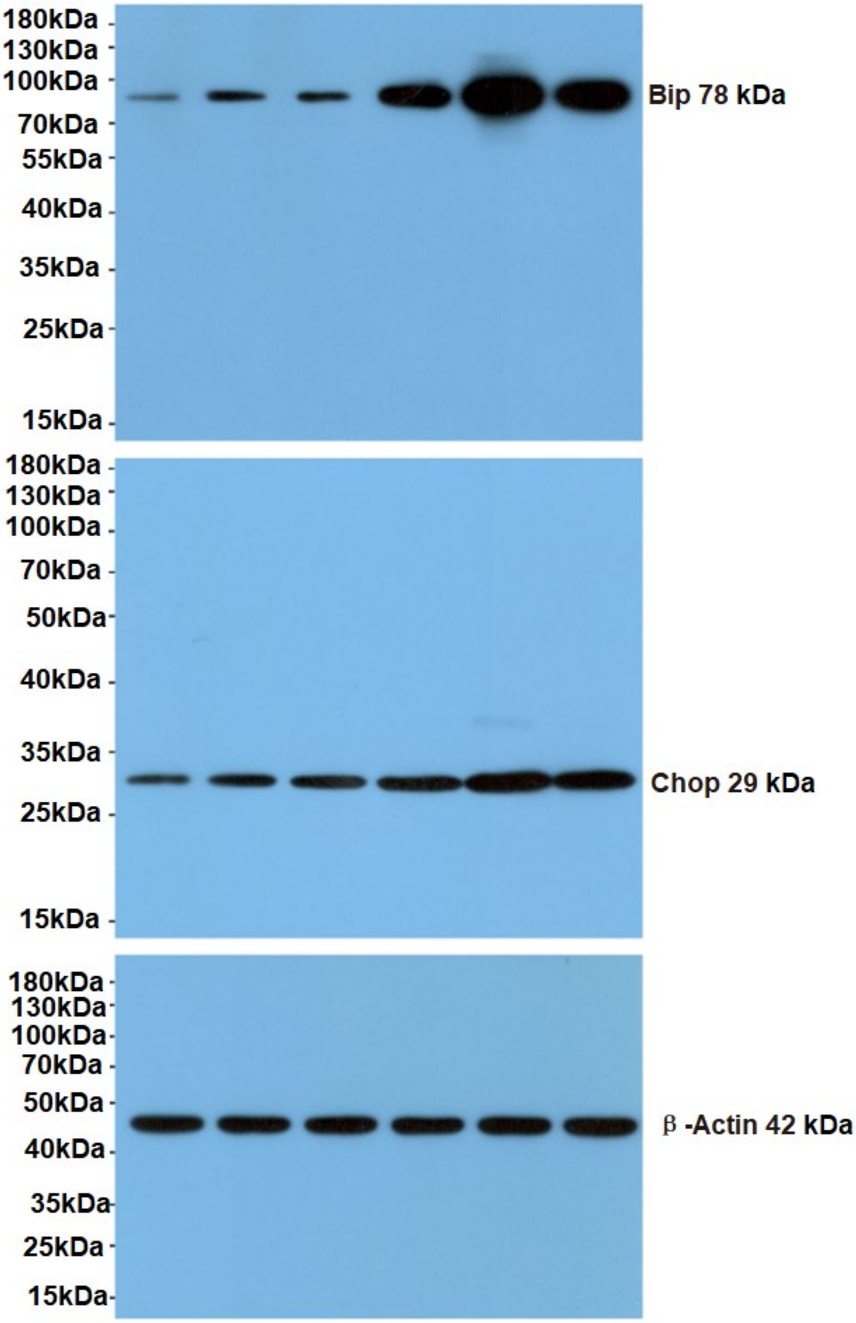

Figure S2: The full length western blots for the Bip and Chop proteins in Fig. 5.

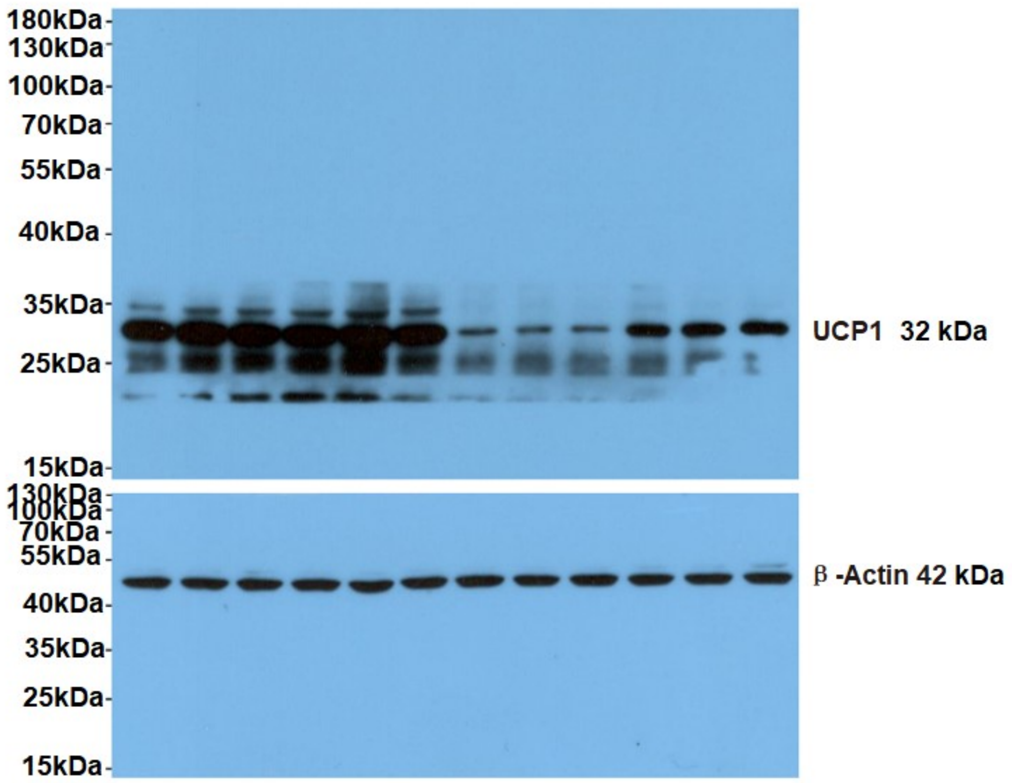

Figure S3: The full length western blots for the UCP1 proteins in Fig.6.
